# Supplementary material for: Elucidating reactive sugar-intermediates by mass spectrometry
Source: Commun Chem. 2025 Mar 7;8:67. doi: 10.1038/s42004-025-01467-5 (PMC11889121; doi:10.1038/s42004-025-01467-5)
Supplement: Supplementary file 2 — SUPPLEMENTAL MATERIAL [file 42004_2025_1467_MOESM2_ESM.pdf]

Table S1. Key features of workflows for computing IR spectra and relative free energies of cationic glycosyl structures using quantum chemistry.

Target	Quantum chemical method	Scale	IR range (cm^{-1})	Temperature	Conformer search
4,6-*O*-Benzylidene glycosyl cations[1]	PBE0/6-311+G(d,p)	0.965	800-1800	90 K	GFN2-xTB (CREST)
Acetylated and benzylated glycosyl cations[2]	PBE+vdW^{TS}/light basis set<br>PBE0-D3/6-311+G(d,p)<br>RIMP2 (def2-TZVPP, def2-QZVPP, def2-QZVPP/C)	0.965	1000-1800	78 K	FAFOOM
Septanosyl ferrier cations[3]	PBE0+D3/6-31G(d)<br>PBE0+D3/6-311+G(d,p)<br>DLPNO-CCSD(T)/Def2-TZVPP	0.965	1000-1800	90 K	GFN2-xTB, GFN-FF (CREST), OPLSe forcefield (Schrödinger Maestro)
Acyl protected galactosyl cations[4]	1. PBE+vdW^{TS}/light basis set<br>PBE0+D3/6-311+G(d,p)<br>DLPNO-CCSD(T)/Def2-TZVPP	0.965	1000-1800	90 K	FAFOOM
Fluorinated and non-fluorinated benzoylated glycosyl cations protected with Fmoc or benzyl groups[5]	PBE/def2-SVP<br>PBE0+D3/6-311+G(d,p)	0.965	1000-1800	90 K	FAFOOM
Ferrier glycosyl cation[6]	PBE+vdW^{TS}/light basis set<br>PBE0+D3/6-311+G(d,p)	0.965	1000-1800	90 K	FAFOOM
2-*O*-Acetyl-glycopyranose[7]	PBE+vdW^{TS}/light basis set<br>PBE0+MBD/tight basis set<br>PBE0+D3/6-311+G(d,p)<br>RIMP2 (def2-TZVPP, def2-QZVPP, def2-QZVPP/C)	0.965	800-1800	-	FAFOOM<br>FENAOC
Uronic acid glycosyl cations[8]	PM6<br>B3LYP/6-31++G(d,p)<br>MP2/6-311++G(2d,2p)	0.975	750-1900	-	DGA<br>MMFF94
Acetylated and methylated glycosyl cations[9]	B3LYP/6-31++G(d, p)<br>MP2/6-311++G(2d, 2p)	0.98	700-1850	-	MMM annealing (AMBER 12)
Glycosyl cations[10]	PM6<br>B3LYP/6-31++G(d,p)<br>MP2	0.975	750-1850	298 K	DGA<br>MMFF
2-*O*-Methyl-3-*O*-acetyl-4,6-*O*-ethylidene-glycosyl cations[11]	PM6<br>B3LYP/6-31++G(d,p)	0.975	750-1850	298.15 K	DGA<br>MMFF94
Rhamnosyl cations[12]	PM6<br>B3LYP/6-31++G(d,p)<br>MP2/6-31++G(d,p)	0.975	750-1850	298.15 K	DGA<br>MMFF94
DMNPA-protected glycosyl cations[13]	PM6<br>B3LYP/6−31++G(d,p)<br>MP2/6-311++G(2d,2p)	0.975	600-1850	298.15 K	DGA<br>MMFF94

DGA: distance geometry algorithm, MMM: molecular mechanics minimization, FENAOC: FHI-aims full electron numerical atomic orbitals code

References

1. Chang, C.-W. *et al.* Mechanistic insight into benzylidene-directed glycosylation reactions using cryogenic infrared spectroscopy. *Nat. Synth.* **3**, 1377-1384 (2024).
2. Marianski, M. *et al.* Remote Participation during Glycosylation Reactions of Galactose Building Blocks: Direct Evidence from Cryogenic Vibrational Spectroscopy. *Angew. Chem. Int. Ed.* 59, 6166–6171 (2020).
3. Greis, K. *et al.* Characterization and Fate of a Septanosyl Ferrier Cation in the Gas and Solution Phases. *J. Org. Chem.* 88, 5543–5553 (2023).
4. Greis, K. *et al.* The Influence of the Electron Density in Acyl Protecting Groups on the Selectivity of Galactose

Formation. *J. Am. Chem. Soc.* 144, 20258–20266 (2022).

5. Greis, K. *et al.* Neighboring Group Participation of Benzoyl Protecting Groups in C3- and C6-Fluorinated Glucose. *Eur. J. Org. Chem.* 2022, e202200255 (2022).

6. Greis, K. *et al.* Direct Experimental Characterization of the Ferrier Glycosyl Cation in the Gas Phase. *Org. Lett.* 22, 8916–8919 (2020).

7. Mucha, E. *et al.* Unravelling the structure of glycosyl cations via cold-ion infrared spectroscopy. *Nat. Commun.* 9, 4174 (2018).

8. Elferink, H. *et al.* Competing C-4 and C-5-Acyl Stabilization of Uronic Acid Glycosyl Cations. *Chem. - Eur. J.* 28, e202201724 (2022).

9. Elferink, H. *et al.* Direct Experimental Characterization of Glycosyl Cations by Infrared Ion Spectroscopy. *J. Am. Chem. Soc.* 140, 6034–6038 (2018).

10. Hansen, T. *et al.* Characterization of glycosyl dioxolenium ions and their role in glycosylation reactions. *Nat. Commun.* 11, 2664 (2020).

11. Remmerswaal, W. A. *et al.* Anomeric Triflates versus Dioxanium Ions: Different Product-Forming Intermediates from 3-Acyl Benzylidene Mannosyl and Glucosyl Donors. *J. Org. Chem.* 89, 1618–1625 (2024).

12. Moons, P. H. *et al.* Characterization of elusive rhamnosyl dioxanium ions and their application in complex oligosaccharide synthesis. *Nat. Commun.* 15, 2257 (2024).

13. Remmerswaal, W. A. *et al.* Stabilization of Glucosyl Dioxolenium Ions by “Dual Participation” of the 2,2-Dimethyl-2-(ortho-nitrophenyl)acetyl (DMNPA) Protection Group for 1,2-cis-Glucosylation. *J. Org. Chem.* 87, 9139–9147 (2022).
